# *Helicobacter pylori* infection as a risk factor for serum bilirubin change and less favourable lipid profiles: a hospital-based health examination survey

**DOI:** 10.1186/s12879-019-3787-8

**Published:** 2019-02-14

**Authors:** Miao-Miao Zhao, Jeremy Krebs, Xi Cao, Jing Cui, Dong-Ning Chen, Yu Li, Lin Hua, Jim Mann, Jin-Kui Yang

**Affiliations:** 1 Department of Endocrinology, Beijing Diabetes Institute, Beijing Tongren Hospital, Capital Medical University, Beijing, 100730 China; 2 Edgar Diabetes and Obesity Research Centre, University of Otago, Wellington, New Zealand; 3 Physical Examination Department, Beijing Tongren Hospital, Capital Medical University, Beijing, 100730 China; 4 Department of Biostatistics and Bioinformatics, School of Biomedical Engineering, Capital Medical University, Beijing, 100069 China; 5 Department of Medicine, Dunedin School of Medicine, University of Otago, Dunedin, New Zealand

**Keywords:** *Helicobacter pylori*, Serum bilirubin, Metabolic disorders, Lipid metabolism

## Abstract

**Background:**

*Helicobacter pylori* infection is associated with several extragastric conditions including dyslipidemia and metabolic syndrome. This study aimed to investigate additional metabolic parameters associated with *H. pylori* infection in a Chinese population.

**Methods:**

Using a case-control approach we studied 617 subjects with ^13^C-urea breath test (^13^C-UBT) values ≥10‰ who were defined as being positive for *H. pylori* (cases), while 617 sex and age- matched subjects with ^13^C-UBT values ≤1‰ were defined as *H. pylori* negative (controls) in Beijing Tongren Hospital from March 2016 to May 2017. Biochemical parameters including serum bilirubin and lipids were tested.

**Results:**

A total of 1982 subjects participated in this study. The *H. pylori* infected subjects had significantly lower serum direct bilirubin concentrations (2.34 ± 0.38 vs. 2.47 ± 0.90 μmol/L, *P* = 0.008). *H. pylori* infection was independently associated with lower direct bilirubin levels (OR = 1.497, 95% CI =1.121–1.999, *P* = 0.006) or total bilirubin levels (OR = 1.322, 95% CI =1.005–1.738, *P* = 0.046) after adjustment for age, sex, body mass index (BMI), alanine aminotransferase (ALT), aspartate aminotransferase (AST), high-density lipoprotein cholesterol (HDL-C), low density lipoprotein-cholesterol (LDL-C), total cholesterol (TC) and triglycerides(TG). In addition, the *H. pylori* infected subjects had higher LDL-C levels (2.98 ± 0.76 vs. 2.89 ± 0.75 mmol/L, *P* = 0.033) and lower HDL-C levels (1.39 ± 0.37 vs. 1.44 ± 0.41 mmol/L, *P* = 0.044). LDL-C was negatively correlated with direct bilirubin concentration (R = − 0.260, *P* < 0.0001).

**Conclusions:**

Bilirubin has been found to be a potent endogenous antioxidant and negatively associated with metabolic syndrome. Our results suggest that *H. pylori* infection is an independent risk factor for serum bilirubin reduction and less favorable lipid profiles.

**Electronic supplementary material:**

The online version of this article (10.1186/s12879-019-3787-8) contains supplementary material, which is available to authorized users.

## Background

*Helicobacter pylori* infection affects ~ 50% of the world’s population and has been recognized as one of the most common chronic infections in human. [1] The overall prevalence is high in developing countries. *H. pylori* infection cause upper gastrointestinal diseases including gastritis, peptic ulcer disease and also increase the risk of gastric cancer. Interestingly, several studies suggest that *H. pylori* infection may influence the gut microbiome [2–4]. Further, diverse extragastric diseases have been linked to *H. pylori* infection, including dyslipidemia [5], type 2 diabetes [6], insulin resistance [7] and metabolic syndrome [8]. The correlation of *H. pylori* infection and bilirubin levels has not been reported. Nevertheless, *H. pylori* infection appears to play an important role in the development of metabolic disorders in which require further investigations.

In the current study, we aimed to investigate additional metabolic parameters and their clinical impact with regard to *H. pylori* infection in a Chinese population.

## Methods

### Study design and population

We performed a case-control study by selecting subjects who were overtly positive for *H. pylori* as cases and overtly negative controls matched by sex and age. We screened subjects aged 18–79 years who were receiving annual health examinations including ^13^C –urea breath test (UBT) in Beijing Tongren Hospital from March 2016 to May 2017. Subjects with ^13^C-UBT values≥10‰ or ≤ 1‰ were defined as overtly positive (cases) or overtly negative (controls) for *H. pylori*, respectively. Subjects with ^13^C-UBT results between 1‰ and 10‰ were excluded from the study. Subjects in the *H. pylori* positive group were matched 1:1 with age and sex to *H. pylori* negative individuals. After the primary assessment of the baseline characters for all subjects, we further excluded participants with liver and gall bladder diseases (hepatitis, jaundice, cholecystitis, biliary calculus), abnormal liver function (alanine aminotransferase (ALT) or aspartate aminotransferase (AST) > 1.5 times upper normal limit, or bilirubin > twice upper normal limit), abnormal kidney function (Cr > upper normal limit) to better eliminate the potential biases caused by diseases.

### Anthropometric and laboratory measurements

Each subject had anthropometric measurements. Presence of systematic or previous diseases, such as diabetes mellitus (DM), hypertension, hepatitis, jaundice, cholecystitis or biliary calculus were noted. Body mass index (BMI) was measured as weight (kg) divided by height (meters) squared (kg/m^2^). Waist circumference (WC) was measured at the level of the umbilicus in cm. Blood pressure (BP) was measured three times when participants were seated, and the average of the last two measurements was recorded. Blood samples were collected after an overnight fasting for the determination of plasma glucose, glycosylated hemoglobin A1c (HbA1c), total cholesterol (TC), triglycerides (TG), high density lipoprotein cholesterol (HDL-C), low density lipoprotein cholesterol (LDL-C), direct bilirubin, total bilirubin, total bile acids, alkaline phosphatase (ALP), ALT, AST [9], γ-glutamyl transpeptidase (γ-GT), blood urea nitrogen [10], serum creatinine (SCr) and uric acids (UA) concentrations.

### Statistical analysis

Data are presented as the mean ± SD. The baseline characteristics of subjects were compared using the chi-squared test for categorical variables and the Student *t*-test for continuous variables. Distribution of discrete/qualitative variables was compared by trend chi-square test. Binary logistic regression analysis was used to estimate crude and adjusted odds ratios (ORs) (95% CIs) to assess the risk of bilirubin change associated with *H. pylori* infection. When data were not normally distributed, the correlations of bilirubin and cholesterol were determined by the Spearman correlation coefficient analysis. Calculations were performed using SPSS 24.0 statistical software (SPSS Inc., Chicago, IL, USA), and significance was established at a two-tailed *P* < 0.05.

## Results

### Clinical characteristics of the study population

**Fig. 1:**
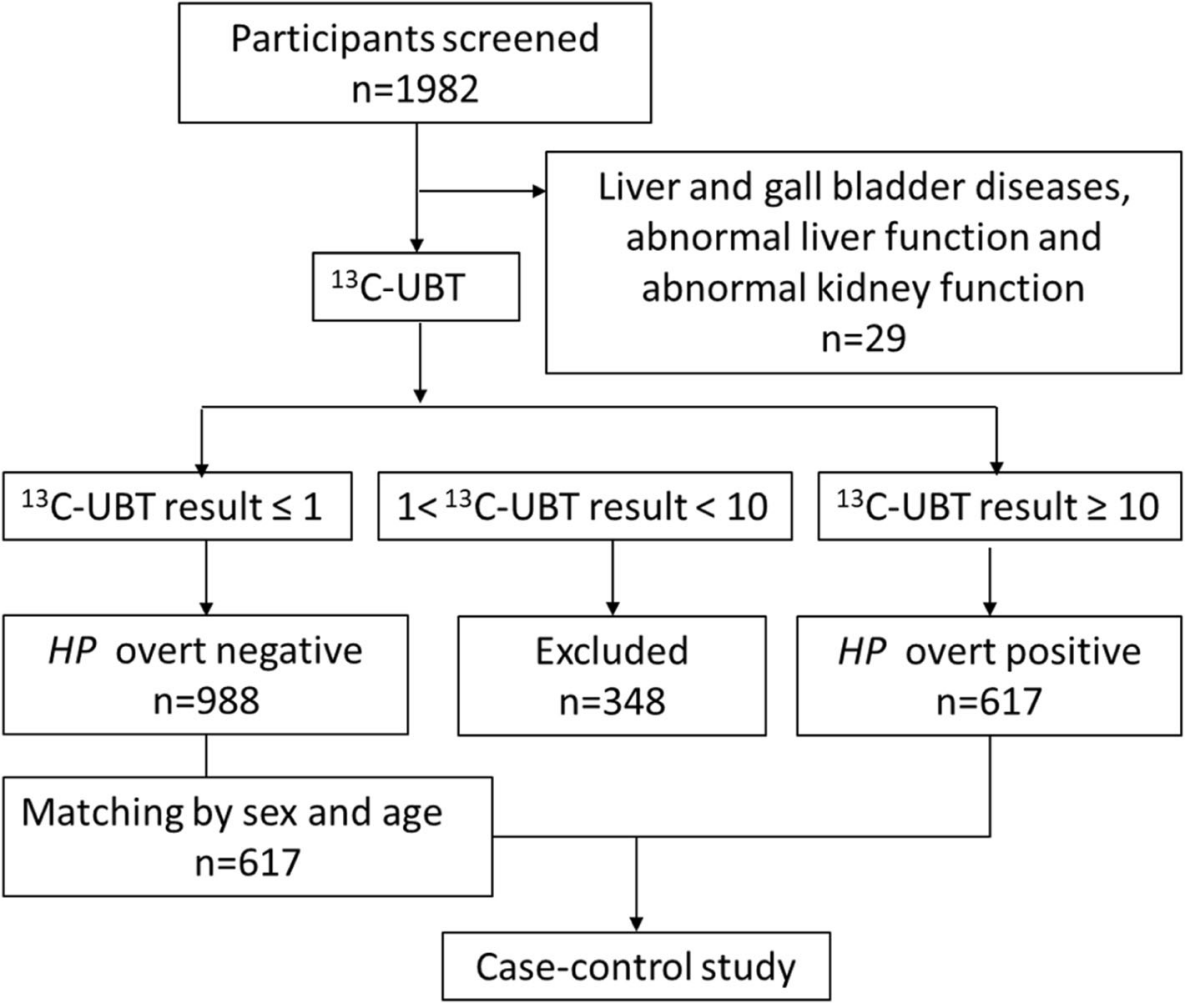
Design and inclusion flow chart of the case-control study. ^13^C-UBT: ^13^C-urea breath tests; *HP*: *H. pylori*

A total of 1982 subjects participated in the health examination including ^13^C -UBT, physical examination and blood tests. Based on selection criteria, 623 subjects were initially defined as cases, 1010 were defined as controls, and 349 were excluded from the study. The primary baseline characters of the two groups are shown in Additional file 1: Table S1. Unexpectedly, our results unveiled an intriguing association between bilirubin decrease and *H. pylori* infection (direct bilirubin 2.50 ± 0.99 vs. 2.37 ± 0.90 μmol/L, *P* = 0.015, total bilirubin 14.49 ± 5.66 vs. 13.87 ± 5.32 μmol/L, *P* = 0.049). We doubted if other disorders may influence the bilirubin levels and caused the inauthentic results, hence, we excluded participants with diseases which could affect bilirubin levels pathologically. According to the specified criteria, 29 patients were excluded from the study. Of the remaining 1953 subjects, 617 (31.6%) overtly positive for *H. pylori* were matched 1:1 with age and sex to 988 (50.6%) overtly negative for *H. pylori*, 348 subjects with ^13^C-UBT results between 1‰ and 10‰ were excluded from the study. Thus, 617 overtly positive *H. pylori* individuals (case group) and 617 sex and age- matched overtly negative *H. pylori* subjects (control group) comprised the case-control study (Fig. 1).

The demographic and biochemical parameters of the cases and the controls are shown in Table 1**.** The case group had significantly less favourable lipid profiles than controls: LDL-C 2.98 ± 0.76 vs. 2.89 ± 0.75 mmol/L (*P* = 0.033), HDL-C 1.39 ± 0.37 vs. 1.44 ± 0.41 mmol/L (*P* = 0.044). Unsurprisingly, the case group also had lower bilirubin levels compared with the control group: direct bilirubin 2.34 ± 0.38 vs. 2.47 ± 0.90 μmol/L (*P* = 0.008), total bilirubin 13.65 ± 4.84 vs. 14.28 ± 5.01 μmol/L (*P* = 0.026). There was no significant difference in other demographic and clinical characteristics.

**Table 1:** Comparison of various clinical characteristics classified by *H. pylori* infection

	*H. pylori* obvious negative (*n* = 617)	*H. pylori* obvious positive (n = 617)	*P* value
Age (years)	41.2 ± 11.7	41.3 ± 11.3	0.910
Female (%)	54.3	54.3	matching
Waist circumference (cm)	80.72 ± 11.33	80.37 ± 11.64	0.601
BMI (kg/m^2^)	24.32 ± 3.78	24.34 ± 3.89	0.943
SBP (mmHg)	118.0 ± 13.4	116.8 ± 12.9	0.103
DBP (mmHg)	75.2 ± 9.1	74.9 ± 9.0	0.465
Hypertension (%)	15.9	13.8	0.334
Fasting blood glucose (mmol/l)	5.37 ± 1.54	5.34 ± 1.13	0.705
Diabetes (%)	5.0	6.2	0.389
Total cholesterol (mmol/l)	4.90 ± 0.88	4.98 ± 0.89	0.142
Triglycerides (mmol/l)	1.40 ± 0.99	1.43 ± 1.22	0.651
HDL-C (mmol/l)	1.44 ± 0.41	1.39 ± 0.37	**0.044**
LDL-C (mmol/l)	2.89 ± 0.75	2.98 ± 0.76	**0.033**
Direct bilirubin (μmol/l)	2.47 ± 0.90	2.34 ± 0.83	**0.008**
Total bilirubin (μmol/l)	14.28 ± 5.01	13.65 ± 4.84	**0.026**
Total bile acids (μmol/L)	3.27 ± 2.20	3.21 ± 2.35	0.657
Alkaline phosphatase (U/L)	73.68 ± 20.93	74.14 ± 21.62	0.706
ALT (U/L)	22.19 ± 16.37	22.33 ± 18.18	0.883
AST (U/L)	22.14 ± 10.23	22.09 ± 11.04	0.932
γ-GT (U/L)	28.55 ± 23.05	28.31 ± 25.81	0.864
BUN (mmol/L)	4.73 ± 1.22	4.68 ± 1.15	0.442
Serum creatinine (μmol/L)	65.88 ± 13.61	66.56 ± 14.35	0.391
Uric acid (μmol/L)	331.87 ± 86.34	330.67 ± 88.87	0.809

*SBP* systolic blood pressure, *DBP* diastolic blood pressure

Data are mean ± SD; Student *t*-test

### Association between serum bilirubin levels and *H. pylori* infection

**Fig. 2:**
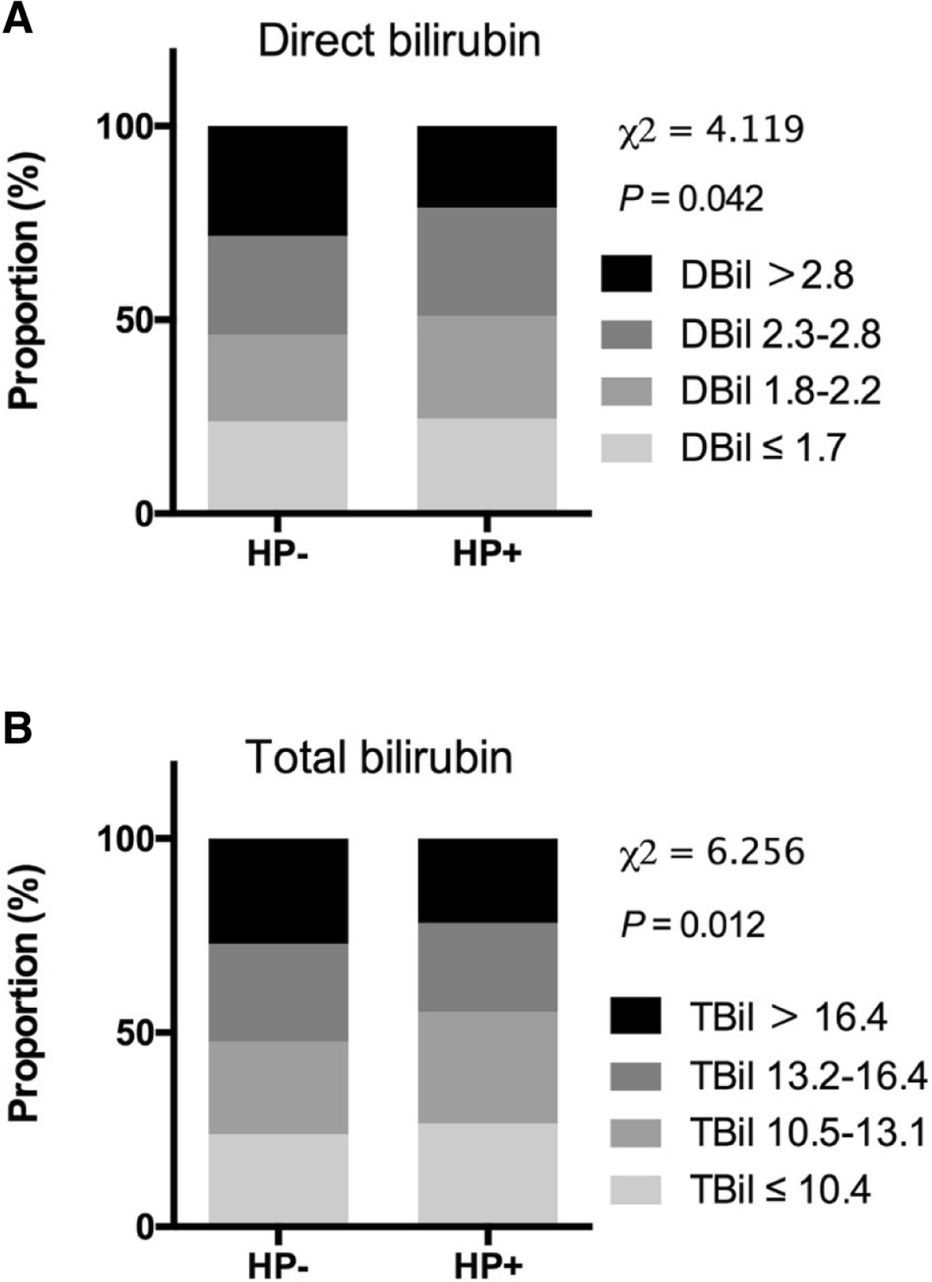
The association between serum bilirubin levels and *H. pylori* infection. **a** Proportion of subjects according to the quartiles of direct bilirubin. The percentages of each grade from higher to lower direct bilirubin levels were 28.4, 25.4, 22.4, 23.8% respectively in *H. pylori* overt negative group and 21.1, 27.9, 26.5, 24.5% respectively in *H. pylori* overt positive group. **b** Proportion of subjects according to the quartiles of total bilirubin. The percentages of each grade from higher to lower total bilirubin levels were 27.2, 25.1, 24.0, 23.7% respectively in *H. pylori* overt negative group and 21.8, 22.9, 28.7, 26.6% respectively in *H. pylori* overt positive group. HP, *H. pylori*

To further investigate the correlation between *H. pylori* infection and bilirubin levels, subjects from both groups were assigned to 4 grades based on quartiles of serum bilirubin concentrations and the proportions of each grade in case and control groups are shown in Fig. 2. In the *H. pylori* positive group, the proportion of subjects with direct bilirubin levels in the highest quart (> 2.8 μmol/L) was smaller than that of the *H. pylori* negative group (21.1% vs 28.4%) (Trend χ^2^ = 4.119, *P* = 0.042). The same trend was also observed in subjects assigned by total bilirubin level (Trend χ^2^ = 6.256, *P* = 0.012). This suggests that *H. pylori* infection is associated with both direct and total bilirubin reduction.

Similar trend was observed when subjects were grouped according to quartiles of LDL-C level (Trend χ^2^ = 4.577, *P* = 0.032, data not shown), but was not seen when subjects grouped according to quartiles of HDL-C level (Trend χ^2^ = 1.185, *P* = 0.288, data not shown) although the mean of HDL-C was significant different in *H. pylori* overtly positive and negative groups (Table 1).

### The risk of bilirubin changes according to the infection of *H. pylori*

Given the association between increased serum bilirubin levels within the reference range and better health outcomes (or conversely lower bilirubin concentrations with higher morbidities) [11–16], we assigned subjects into two groups with direct bilirubin (2.8) and total bilirubin (16.4) in the upper distribution quartile. Bilirubin levels below the upper quartile were defined as “bilirubin not increase”. Logistic regression analysis was performed to determine the independence of the association between *H. pylori* infection and risk for “bilirubin not increase”. As shown in Table 2, *H. pylori* infection was associated with higher risk of both direct bilirubin (OR = 1.483, 95% CI =1.143–1.925, *P* = 0.003) and total bilirubin (OR = 1.336, 95% CI =1.030–1.733, *P* = 0.029) “not increase” in univariate analysis. After adjustment for age and sex (model 2), further adjustment for BMI, ALT and AST (model 3), and then additional adjustment for HDL-C, LDL-C, TC and TG (model 4), the ORs remained significant.

**Table 2:** The risk of bilirubin decreases according to the infection of *H. pylori*

	Direct bilirubin			Total bilirubin		
	Odds ratio	95% CI	*P* value	Odds ratio	95% CI	*P* value
Model 1	1.483	1.143–1.925	0.003	1.336	1.030–1.733	0.029
Model 2	1.500	1.151–1.955	0.003	1.352	1.036–1.764	0.026
Model 3	1.503	1.146–1.972	0.003	1.328	1.014–1.739	0.039
Model 4	1.497	1.121–1.999	0.006	1.322	1.005–1.738	0.046

Participants were assigned to two groups according to upper quartile of direct bilirubin (2.8) and total bilirubin (16.4) respectively. Participants with *H. pylori* overt negative were defined as 0 and those with *H. pylori* overt positive were defined as 1. Model 1 is unadjusted. Model 2 is adjusted for age, sex. Model 3 is further adjusted for BMI, ALT and AST. Model 4 is further adjusted for TG, TC, LDL-C, HDL-C

### Correlation between direct bilirubin and cholesterol

**Fig. 3:**
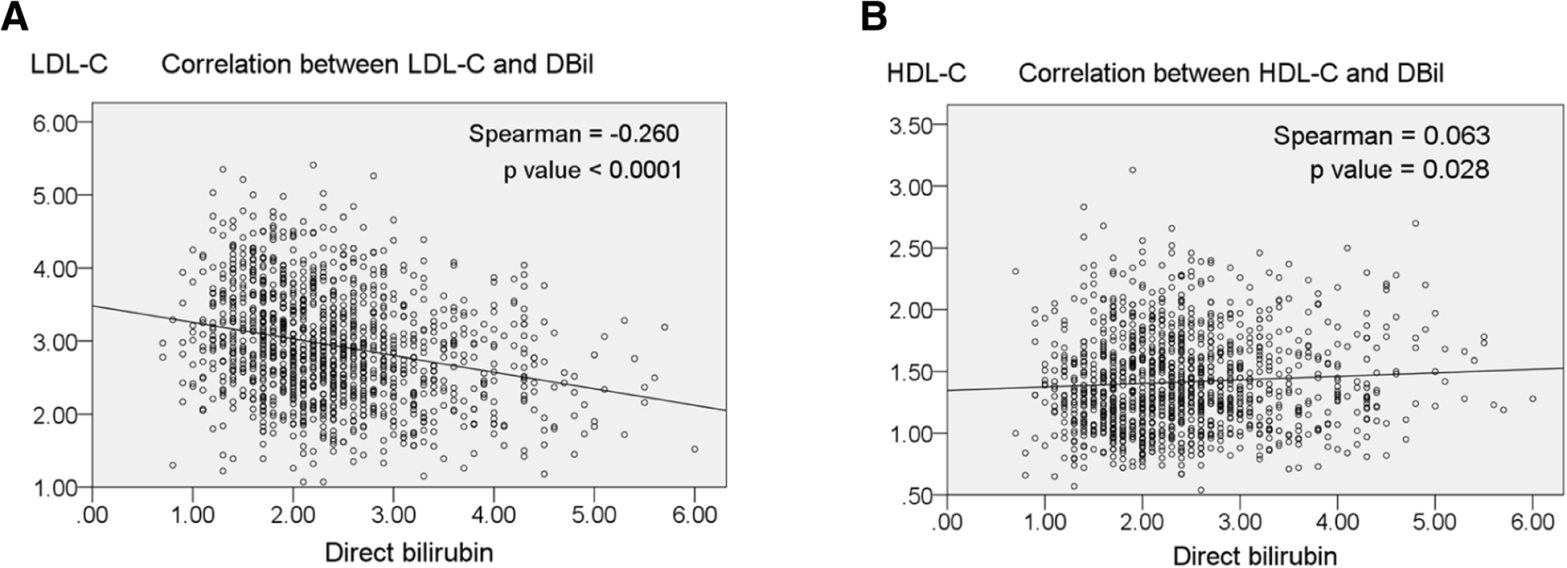
Correlation between direct bilirubin and cholesterol. **a** Correlation between LDL-cholesterol (LDL-C) and direct bilirubin (DBil). **b** Correlation between HDL-cholesterol (HDL-C) and DBil

We found that lipid profiles improved with increasing direct bilirubin levels regardless of *H. pylori* status. LDL-C was negatively correlated with direct bilirubin concentration (R = − 0.260, *P* < 0.0001) (Fig. 3a), while HDL-C was positively correlated with direct bilirubin level. (R = 0.063, *P* = 0.028) (Fig. 3b).

## Discussion

With the sensitivity and specificity exceeding 90%, UBT is often considered as the gold standard test in the diagnosis of *H. pylori* infection [17–19]. However, there is a “grey zone” of uncertainty when UBT values range from 2.0 to 5.0‰ [20, 21]. Fortunately, positive and negative UBT results tend to cluster outside this range [22]. We performed this study by selecting subjects at the extreme ends of the range of ^13^C-UBT values to comprise the study groups. We selected subjects with ^13^C-UBT values ≤1 ‰ and ≥ 10 ‰ to avoid false-positive and false-negative results.

In this case-control study, we found that subjects with high ^13^C-UBT values had lower bilirubin concentrations and less favourable lipid profiles compared those with low ^13^C-UBT values. In addition to being a breakdown product of heme, serum bilirubin is also a powerful antioxidant [23, 24]. High normal concentrations of serum bilirubin correlate with better health outcomes [11–16]. Bilirubin concentrations have been reported as being inversely associated with risk for cardiovascular disease [15, 25], metabolic syndrome [16], diabetes [11], inflammatory disease [13] and some cancers [14]. However, little is known of determinants of bilirubin levels within the reference range. To our knowledge, this study indicates for the first time that *H. pylori* infection may be associated with decreased bilirubin concentrations within the reference range. *H. pylori* infection has been shown to result in chronic inflammation and influence of bile reflux [9, 26], which may at least in part explain the bilirubin changes, but further research relating to possible mechanisms is required.

Our study also found an association between *H. pylori* infection and lipid profiles. Serum LDL-C level was significantly higher and HDL-C significantly lower in *H. pylori* infected subjects. This association was first observed in 1996 in Finnish subjects [27]. Since then, several studies have been performed in different populations. However, the results are still equivocal. Most studies have supported the significant correlation between *H. pylori* infection and elevated lipids levels [5, 10, 28–33]. However Elizalde et al. found that *H. pylori* infection had no influence on blood lipids in 686 *H. pylori*-positive patients before and 3 months after eradication therapy with a low treatment rate (53.6%) [9]. It should be noted that cases and controls were not matched for sex and age which are key influence determinants of serum lipids. Furthermore *H. pylori* infection is associated with a long-term effect on human health [34] and 3 months may not be long enough to observe changes resulting from eradication of *H. pylori.* In our relatively large study, we selected individuals with extreme ^13^C-UBT values to better distinguish the differences associated with *H. pylori* infection and matched the subjects by sex and age. We found that *H. pylori* infected subjects had significantly higher LDL-C and lower HDL-C levels.

Our study also found that decreased direct bilirubin was correlated with adverse lipid profiles, an important cause of cardiovascular disease and a feature of clusters of metabolic disease risk factors. Recent in vivo and in vitro studies suggest that this may due to bilirubin regulation of the fat burning nuclear receptor, PPAR-α and γ levels and thus inhibited lipid accumulation [35, 36]. Given that *H. pylori* infection may influence lipid profiles and that we could not prove a causal relationship between bilirubin and cholesterol levels. It is conceivable that the elevated cholesterol levels may be a result of both *H. pylori* infection and decreased bilirubin concentrations.

Limitations of our study should be acknowledged. First, our study was not a prospective study, so we could not examine the effects of eradication therapy. Comparing bilirubin and lipid levels before and after eradication of *H. pylori* would enable more definitive conclusions. Second, our study was a cross-sectional study. Despite of statistical significance in lipid profiles between groups, we could not assure a real difference in clinical practice. Larger sample size prospective study is still required in the future.

## Conclusion

Our data suggest that *H. pylori* infection may be an independent risk factor for serum bilirubin reduction and adverse lipid profiles. If confirmed, this would provide further evidence for the importance of diagnosis and eradication of *H. pylori* infection.

## Additional files

Additional file 1: **Table S1.** Primary baseline characteristics classified by *H. pylori* infection. SBP, systolic blood pressure; DBP, diastolic blood pressure. Data are mean ± SD; Student *t*-test. (DOCX 365 kb)

Additional file 2: Database. Primary database of this study. (SAV 181 kb)

## Abbreviations

ALP: Alkaline phosphatase
ALT: Alanine aminotransferase
AST: Aspartate aminotransferase
BMI: Body mass index
BP: Blood pressure
DM: Diabetes mellitus
HbA1c: Glycosylated hemoglobin A1c
HDL-C: High-density lipoprotein cholesterol
LDL-C: Low density lipoprotein-cholesterol
OR: Odds ratio
SCr: Serum creatinine
TC: Total cholesterol
TG: Triglycerides
UA: Uric acids
UBT: Urea breath test
WC: Waist circumference
γ-GT: γ-glutamyl transpeptidase

## References

[1] Kibru D, Gelaw B, Alemu A, Addis Z. Helicobacter pylori infection and its association with anemia among adult dyspeptic patients attending Butajira hospital, Ethiopia. BMC Infect Dis*,*14*,*1(2014-12-09). 2014;14(1):656. 10.1186/s12879-014-0656-3 PMC4264248 25487159

[2] Heimesaat MM, Fischer A, Plickert R, Wiedemann T, Loddenkemper C, Gobel UB, Bereswill S, Rieder G (2014). Helicobacter pylori induced gastric immunopathology is associated with distinct microbiota changes in the large intestines of long-term infected Mongolian gerbils. PLoS One.

[3] Maldonado-Contreras A, Goldfarb KC, Godoy-Vitorino F, Karaoz U, Contreras M, Blaser MJ, Brodie EL, Dominguez-Bello MG (2011). Structure of the human gastric bacterial community in relation to helicobacter pylori status. ISME J.

[4] Yin YN, Wang CL, Liu XW, Cui Y, Xie N, Yu QF, Li FJ, Lu FG (2011). Gastric and duodenum microflora analysis after long-term helicobacter pylori infection in Mongolian gerbils. Helicobacter.

[5] Satoh H, Saijo Y, Yoshioka E, Tsutsui H (2010). Helicobacter pylori infection is a significant risk for modified lipid profile in Japanese male subjects. J Atheroscler Thrombosis.

[6] Hsieh MC, Wang SS, Hsieh YT, Kuo FC, Soon MS, Wu DC (2013). Helicobacter pylori infection associated with high HbA1c and type 2 diabetes. Eur J Clin Investig.

[7] Zhou X, Liu W, Gu M, Zhou H, Zhang G (2015). Helicobacter pylori infection causes hepatic insulin resistance by the c-Jun/miR-203/SOCS3 signaling pathway. J Gastroenterol.

[8] Refaeli R, Chodick G, Haj S, Goren S, Shalev V, Muhsen K. Relationships of H. Pylori infection and its related gastroduodenal morbidity with metabolic syndrome: a large cross-sectional study. Sci Rep. 2018;8(1). 10.1038/s41598-018-22198-9 PMC5840265 29511278

[9] Elizalde JI, Piqué JM, Moreno V, Morillas JD, Elizalde I, Bujanda L, Argila CMD, Cosme A, Castiella A, Ros E (2002). Influence of helicobacter pylori infection and eradication on blood lipids and fibrinogen. Aliment Pharmacol Ther.

[10] Kucukazman M, Yavuz B, Sacikara M, Asilturk Z, Ata N, Ertugrul DT, Yalcin AA, Yenigun EC, Kizilca G, Okten H (2009). The relationship between updated Sydney system score and LDL cholesterol levels in patients infected with helicobacter pylori. Dig Dis Sci.

[11] Cheriyath P, Gorrepati VS, Peters I, Nookala V, Murphy ME, Srouji N, Fischman D (2010). High Total bilirubin as a protective factor for diabetes mellitus: an analysis of NHANES data from 1999 - 2006. J Clin Med Res.

[12] Curtin JJ, Fairchild BA (2003). Alcohol and cognitive control: implications for regulation of behavior during response conflict. J Abnorm Psychol.

[13] Fischman D, Valluri A, Gorrepati VS, Murphy ME, Peters I, Cheriyath P (2010). Bilirubin as a protective factor for rheumatoid arthritis: an NHANES study of 2003 - 2006 data. J Clin Med Res.

[14] Horsfall LJ, Rait G, Walters K, Swallow DM, Pereira SP, Nazareth I, Petersen I (2011). Serum bilirubin and risk of respiratory disease and death. JAMA.

[15] Novotný L, Vítek L (2003). Inverse relationship between serum bilirubin and atherosclerosis in men: a meta-analysis of published studies. Exp Biol Med.

[16] Wu Y, Li M, Xu M, Bi Y, Li X, Chen Y, Ning G, Wang W (2011). Low serum total bilirubin concentrations are associated with increased prevalence of metabolic syndrome in Chinese. J Diab.

[17] Graham DY, Klein PD (2000). Accurate diagnosis of helicobacter pylori. 13C-urea breath test. Gastroenterol Clin N Am.

[18] Parente F, Bianchi PG (2001). The (13)C-urea breath test for non-invasive diagnosis of helicobacter pylori infection: which procedure and which measuring equipment?. Eur J Gastroenterol Hepatol.

[19] Gisbert JP, Pajares JM, BMC Infectious Diseases (2004). Review article: 13C-urea breath test in the diagnosis of helicobacter pylori infection -- a critical review. Aliment Pharmacol Ther.

[20] Mion F, Rosner G, Rousseau M, Minaire Y (1997). 13C-urea breath test for helicobacter pylori: cut-off point determination by cluster analysis. Clin Sci.

[21] Peng NJ, Pingi H, Shuicheng L, Hueihwa T, Huang WK, Dawguey T, Luping G, Ginho L, Lin CK, Chichang T (2000). A 15-minute [13C]-urea breath test for the diagnosis of helicobacter pylori infection in patients with non-ulcer dyspepsia. J Gastroenterol Hepatol.

[22] Gisbert JP, Olivares D, Jimenez I, Pajares JM (2006). Long-term follow-up of 13 C-urea breath test results after helicobacter pylori eradication: frequency and significance of borderline δ 13 CO 2 values. Aliment Pharmacol Ther.

[23] Rizzo AM, Berselli P, Zava S, Montorfano G, Negroni M, Corsetto P, Berra B (2010). Endogenous antioxidants and radical scavengers. Oxygen Transport to Tissue XXXIII.

[24] Stocker R, Yamamoto Y, Mcdonagh AF, Glazer AN, Ames BN (1987). Bilirubin is an antioxidant of possible physiological importance. Science.

[25] Perlstein TS, Pande RL, Beckman JA, Creager MA (2008). Serum total bilirubin level and prevalent lower-extremity peripheral arterial disease: National Health and nutrition examination survey (NHANES) 1999 to 2004. Arterioscler Thromb Vasc Biol.

[26] Netzer P, Gut A, Brundler R, Gaia C, Halter F, Inauen W (2001). Influence of pantoprazole on oesophageal motility, and bile and acid reflux in patients with oesophagitis. Aliment Pharmacol Ther.

[27] Niemelä S, Karttunen T, Korhonen T, Läärä E, Karttunen R, Ikäheimo M, Kesäniemi YA (1996). Could helicobacter pylori infection increase the risk of coronary heart disease by modifying serum lipid concentrations?. Heart.

[28] Laurila A, Bloigu A, Näyhä S, Hassi J, Leinonen M, Saikku P (1999). Association of Helicobacter pylori infection with elevated serum lipids. Atherosclerosis.

[29] Chimienti G, Russo F, Lamanuzzi BL, Nardulli M, Messa C, Di LA CM, Giannuzzi V, Pepe G (2003). Helicobacter pylori is associated with modified lipid profile: impact on lipoprotein(a). Clin Biochem.

[30] Georges JL, Rupprecht HJ, Blankenberg S, Poirier O, Bickel C, Hafner G, Nicaud V, Meyer J, Cambien F, Tiret L (2003). Impact of pathogen burden in patients with coronary artery disease in relation to systemic inflammation and variation in genes encoding cytokines. Am J Cardiol.

[31] Karpouza A, Samouilidou E, Karagiannis S, Kostopoulou V, Sotiropoulou M, Roma E, Petraki K, Michopoulos S (2008). Patients with duodenal ulcer have lower levels of serum cholesterol compared to other dyspeptic patients independently of helicobacter pylori status. Scand J Gastroenterol.

[32] Jia EZ, Zhao FJ, Hao B, Zhu TB, Wang LS, Chen B, Cao KJ, Huang J, Ma WZ, Yang ZJ: Helicobacter pylori infection is associated with decreased serum levels of high density lipoprotein, but not with the severity of coronary atherosclerosis. Lipids Health Dis 2009; 8(1):59–59. 10.1186/1476-511X-8-59 PMC2808301 20030806

[33] Kim HL, Han HJ, Park IY, Jin MC, Ji SK, Min KW (2011). Helicobacter pylori infection is associated with elevated low density lipoprotein cholesterol levels in elderly Koreans. J Korean Med Sci.

[34] Buzás GM (2014). Metabolic consequences of helicobacter pylori infection and eradication. World J Gastroenterol.

[35] Liu J, Dong H, Zhang Y, Cao M, Song L, Pan Q, Bulmer A, Adams DB, Dong X, Wang H (2016). Corrigendum: bilirubin increases insulin sensitivity by regulating cholesterol metabolism, Adipokines and PPARγ levels. Sci Rep.

[36] Stec DE, Kezia J, Trabbic CJ, Amarjit L, Hankins MW, Justin B (2016). HTD: bilirubin binding to PPARα inhibits lipid accumulation. PLoS One.

