# Supplementary figures and images for: Helicobacter pylori infection as a risk factor for serum bilirubin change and less favourable lipid profiles: a hospital-based health examination survey
Source: BMC Infect Dis. 2019 Feb 14;19:157. doi: 10.1186/s12879-019-3787-8 (PMC6376701; doi:10.1186/s12879-019-3787-8)

**
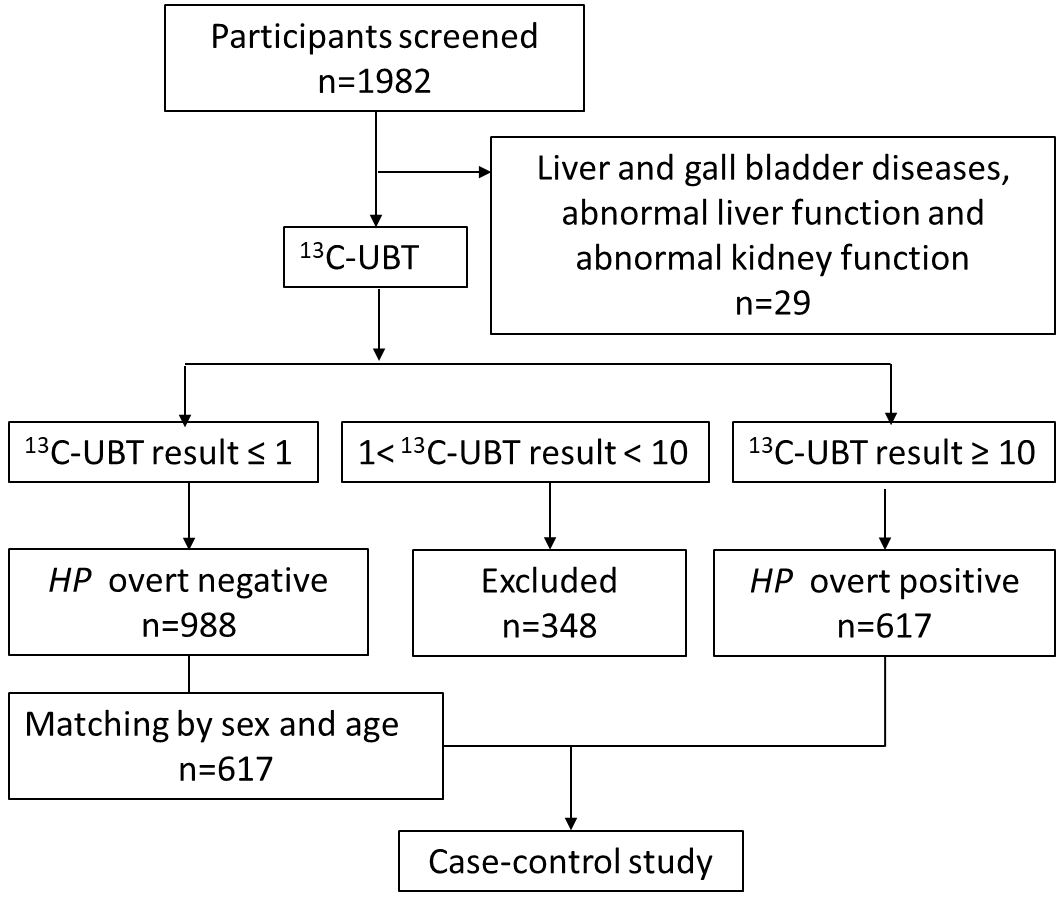
**

**
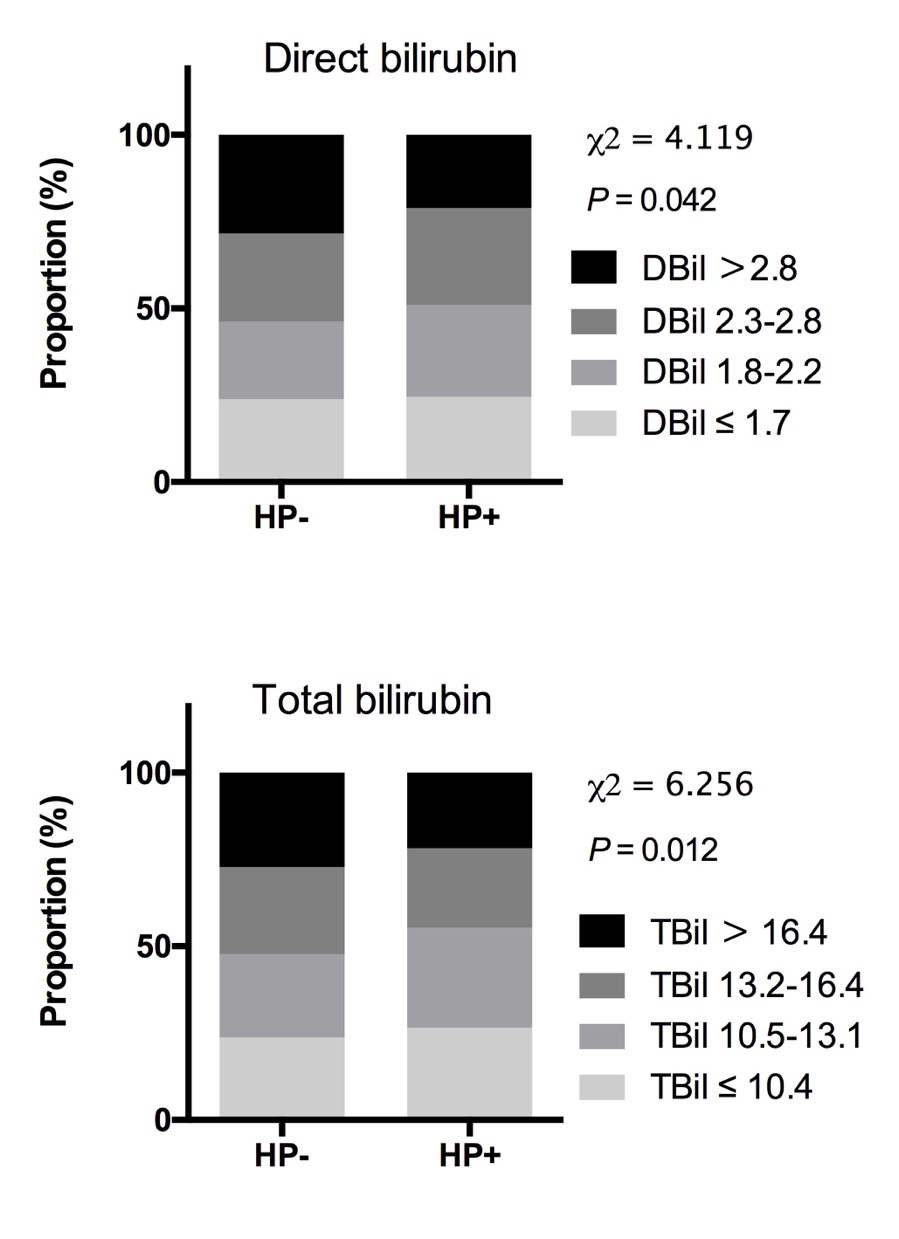
 A**

**B**

A B

**
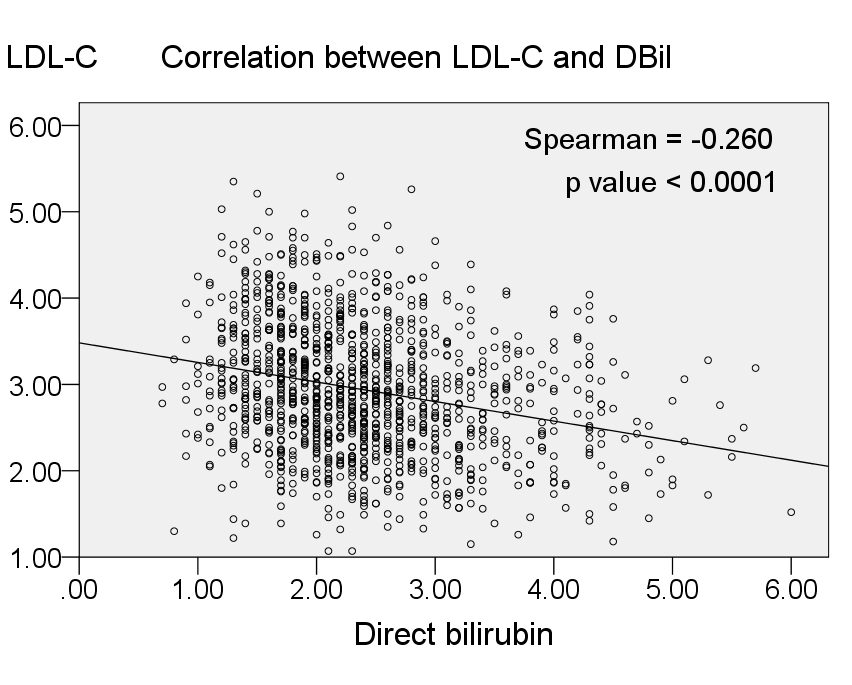

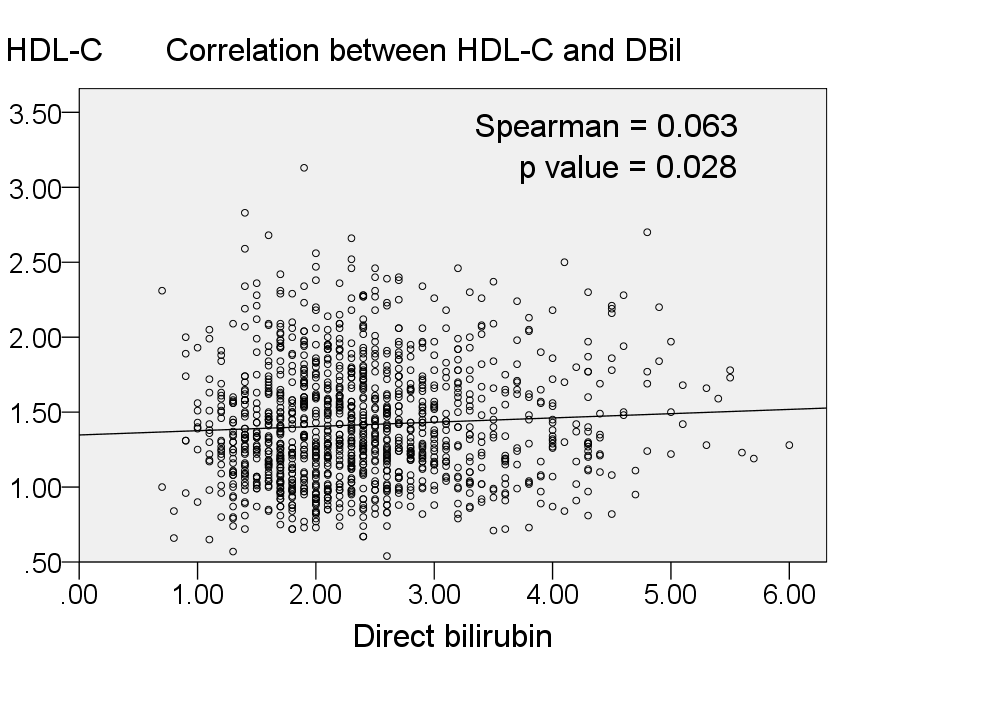
**

Supplement: Supplementary file 1 — Table S1. Primary baseline characteristics classified by H. pylori infection. SBP, systolic blood pressure; DBP, diastolic blood pressure. Data are mean ± SD; Student t-test. (DOCX 365 kb) [file 12879_2019_3787_MOESM1_ESM.docx]
